# A combined cell and growth factor delivery for the repair of a critical size tibia defect using biodegradable hydrogel implants

**DOI:** 10.1002/term.3285

**Published:** 2022-02-04

**Authors:** Talia Cohen, Olga Kossover, Eli Peled, Tova Bick, Lena Hasanov, Tan Tuan Chun, Simon Cool, Dina Lewinson, Dror Seliktar

**Affiliations:** ^1^ The Bruce Rappaport Faculty of Medicine Technion–Israel Institute of Technology Haifa Israel; ^2^ Faculty of Biomedical Engineering Technion–Israel Institute of Technology Haifa Israel; ^3^ Department of Orthopedic Surgery Rambam Medical Center Haifa Israel; ^4^ The Institute of Research of Bone Healing the Rambam Healthcare Campus Haifa Israel; ^5^ Glycotherapeutics Group Institute of Molecular and Cell Biology A*STAR Singapore Singapore

**Keywords:** biomaterials, bone morphogenic protein, growth factors, scaffolds, tissue engineering

## Abstract

The ability to repair critical‐sized long‐bone injuries using growth factor and cell delivery was investigated using hydrogel biomaterials. Physiological doses of the recombinant human bone morphogenic protein‐2 (rhBMP2) were delivered in a sustained manner from a biodegradable hydrogel containing peripheral human blood‐derived endothelial progenitor cells (hEPCs). The biodegradable implants made from polyethylene glycol (PEG) and denatured fibrinogen (PEG‐fibrinogen, PF) were loaded with 7.7 μg/ml of rhBMP2 and 2.5 × 10^6^ cells/ml hEPCs. The safety and efficacy of the implant were tested in a rodent model of a critical‐size long‐bone defect. The hydrogel implants were formed ex‐situ and placed into defects in the tibia of athymic nude rats and analyzed for bone repair after 13 weeks following surgery. The hydrogels containing a combination of 7.7 μg/ml of rhBMP2 and 2.5 × 10^6^ cells/ml hEPCs were compared to control hydrogels containing 7.7 μg/ml of rhBMP2 only, 2.5 × 10^6^ cells/ml hEPCs only, or bare hydrogels. Assessments of bone repair include histological analysis, bone formation at the site of implantation using quantitative microCT, and assessment of implant degradation. New bone formation was detected in all treated animals, with the highest amounts found in the treatments that included animals that combined the PF implant with rhBMP2. Moreover, statistically significant increases in the tissue mineral density (TMD), trabecular number and trabecular thickness were observed in defects treated with rhBMP2 compared to non‐rhBMP2 defects. New bone formation was significantly higher in the hEPC‐treated defects compared to bare hydrogel defects, but there were no significant differences in new bone formation, trabecular number, trabecular thickness or TMD at 13 weeks when comparing the rhBMP2 + hEPCs‐treated defects to rhBMP2‐treated defects. The study concludes that the bone regeneration using hydrogel implants containing hEPCs are overshadowed by enhanced osteogenesis associated with sustained delivery of rhBMP2.

## 1 INTRODUCTION

Autologous bone grafting is the most prevalent intervention in major long‐bone traumatic injuries that necessitate de novo bone repair. A number of critical deficiencies of this technique, including donor site morbidity, increased pain, and limited bone graft availability, have spawned continued research into alternative methods of bone repair in critical‐sized defects (Park, 2011). Recombinant human bone morphogenic protein‐2 (rhBMP2) stands out as highly efficacious among the numerous commercially available products for this clinical indication (Barrilleaux et al., 2006; Ben‐David, Kizhner, Livne, et al., 2010; Ben‐David, Kizhner, Kohler, et al., 2010; Bongio et al., 2010; Dubruel et al., 2007; Martino et al., 2011; Peled et al., 2007; Srouji et al., 2010; Srouji et al., 2006; Srouji et al., 2005; Zhang et al., 2018). Since receiving approval for spinal fusion from the Federal Drug Administration (FDA) in 2002 (Axelrad & Einhorn, 2009; Mont et al., 2004), rhBMP2 has also been used off‐label for other clinical indications, including the repair of long‐bone segmental defects. A major shortcoming of the rhBMP2 approach has been the safety concerns associated with the use of supraphysiological doses of growth factor placed in the device, which can cause adverse effects and pose a risk to patients (Carragee et al., 2011; Schmidmaier et al., 2008; Shields et al., 2006). For example, INFUSE® is a combination device that contains 1.5 mg/ml of rhBMP2 in a porous resorbable collagen sponge. This high dosage of rhBMP2 has been shown to be instrumental in osteogenesis and bone metabolism (Reddi, 1997), but an improved delivery strategy for the growth factor can reduce overall dosage and thereby mitigate some of the primary risks of this approach, include immunological reactions, heterotopic bone formation and edema (Gottfried & Dailey, 2008; James et al., 2016; Villavicencio et al., 2005). Sustained‐release strategies for low‐dose rhBMP2 delivery have been proposed to alleviate the dangers of supraphysiological levels of this potent factor. Much of these efforts are premised on engineered biomaterial delivery strategies.

Cell therapy has similarly been applied as an alternative to off‐the‐shelf commercial products for bone repair indications, namely non‐union long bone defects. The vast majority of cell‐based strategies employ autologous mesenchymal stem cells (MSCs) harvested directly from bone marrow or other tissue origins, including placenta, adipose tissue and umbilical cord blood. MSCs have shown osteogenic differentiation potential in a vast number of experimental studies. Importantly, MSCs have shown excellent potential for long‐bone repair of non‐union fractures in clinical trials. The major disadvantage of tissue‐derived MSCs for bone repair is donor site morbidity. As a possible alternative to MSCs, osteogenic progenitor cells derived from peripheral blood provide an abundant source of autologous cells while addressing the concerns associated with cell harvesting. Kuznetsov et al. (2001) first identified circulating progenitor cells with osteogenic potential in peripheral blood. These cells have been shown to differentiate into endothelial progenitor cells (EPCs) and cells with osteogenic markers (Asahara et al., 1997; Gossl et al., 2008). The EPCs are mainly involved in neovascularization, vasculogenesis and spontaneous angiogenesis associated with tissue repair processes (Fuchs et al., 2009; Hur et al., 2004; Luttun et al., 2002). It is with this intent that EPCs have been applied pre‐clinically for the repair of critical‐sized long‐bone defects in sheep and rodent models (Atesok et al., 2010; Rozen et al., 2009; Seebach et al., 2010). Given the encouraging pre‐clinical results using cell‐based therapies for bone repair, there is a need for biocompatible and biodegradable scaffolds that can deliver these cells to the target site. In addition, the ideal scaffold material should function to retain the cells in their proper phenotype and sustain the bone repair processes in the segmental defects for several weeks.

One of the most effective delivery strategies for either growth factors or cell therapy is using hydrogel biomaterial scaffolds (Ciocci et al., 2018). These scaffolds are made from either synthetic or biological polymeric constituents and are characterized by their large water content and a nano‐scale mesh size (Seliktar, 2012). They can be designed to be biodegradable by cell‐mediated proteolysis. The nano‐porosity of these materials makes them ideal for entrapping growth factors or cells for days and weeks and even synchronize their degradation with the release of the factor or liberation of the transplanted cell population (Cohen et al., 2022; Lev & Seliktar, 2018; Peled et al., 2007; Seliktar, 2005). Additionally, the biomaterial should not obstruct bone regeneration and should locally stimulate the formation of new bone only at the site of implantation by using an osteoinductive formulation that includes either osteogenic progenitor cells, or rhBMP2, or a similar bone growth factor (Liu et al., 2004; Park, 2011; Schmidmaier et al., 2008). Hydrogels that were developed for growth factors or cell delivery based on biodegradation of the implant have been successfully applied for bone regeneration in both maxillofacial and long bone defects (Martino et al., 2011; Xu et al., 2011). Recently, we and others developed hydrogels with the aim of sustained release of rhBMP2 using less than 50 μg/ml of the bioactive factor from hydrogels (Kossover et al., 2020). These biodegradable and bioactive implants demonstrated excellent bone repair capabilities (Ben‐David et al., 2013; Rufaihah & Seliktar, 2016; Yamamoto et al., 2006; Yang et al., 2010).

With the initial success of bone repair hydrogel implants evident based on excellent pre‐clinical outcomes, the need for further enhancement of the bone repair process has taken center stage in the development efforts of future technologies. It is with this aim that we now examined a combination strategy that provides a hydrogel implant with both sustained rhBMP2 release and osteogenic cell therapy using human peripheral blood‐derived EPCs (hEPCs). We formulated a biodegradable hydrogel scaffold for bone repair made from PEG‐fibrinogen (PF; Gonen‐Wadmany et al., 2011), which has been shown previously to be beneficial for treating segmental bone injuries in the rat model when loaded with 7.7 μg/ml of rhBMP2 (Ben‐David et al., 2013; Kossover et al., 2020; Peled et al., 2007). The osteogenic efficacy of the hEPCs was now explored together with the coordinated biodegradation of the implant and the subsequent release of the rhBMP2 using a non‐union long‐bone segmental defect model in the immune‐deficient rat. We examined the ability of PF hydrogels with and without 2.5 × 10^6^ cells/ml hEPCs and/or 7.7 μg/ml rhBMP2 (i.e., 0.27 μg of rhBMP2 in 35 μl total implant volume per defect) to promote new bone formation in a 5‐mm tibial defect. Assessments of bone repair include histological analysis, x‐ray imaging, and quantitative microCT (μCT) analysis. Using this multi‐factorial experimental design, we aim to provide further insight into any potential benefit achieved with a combined cell therapy, growth factor release approach to bone regeneration.

## 2 MATERIALS AND METHODS

### 2.1 Human endothelial progenitor cell isolation

**FIGURE 1:**
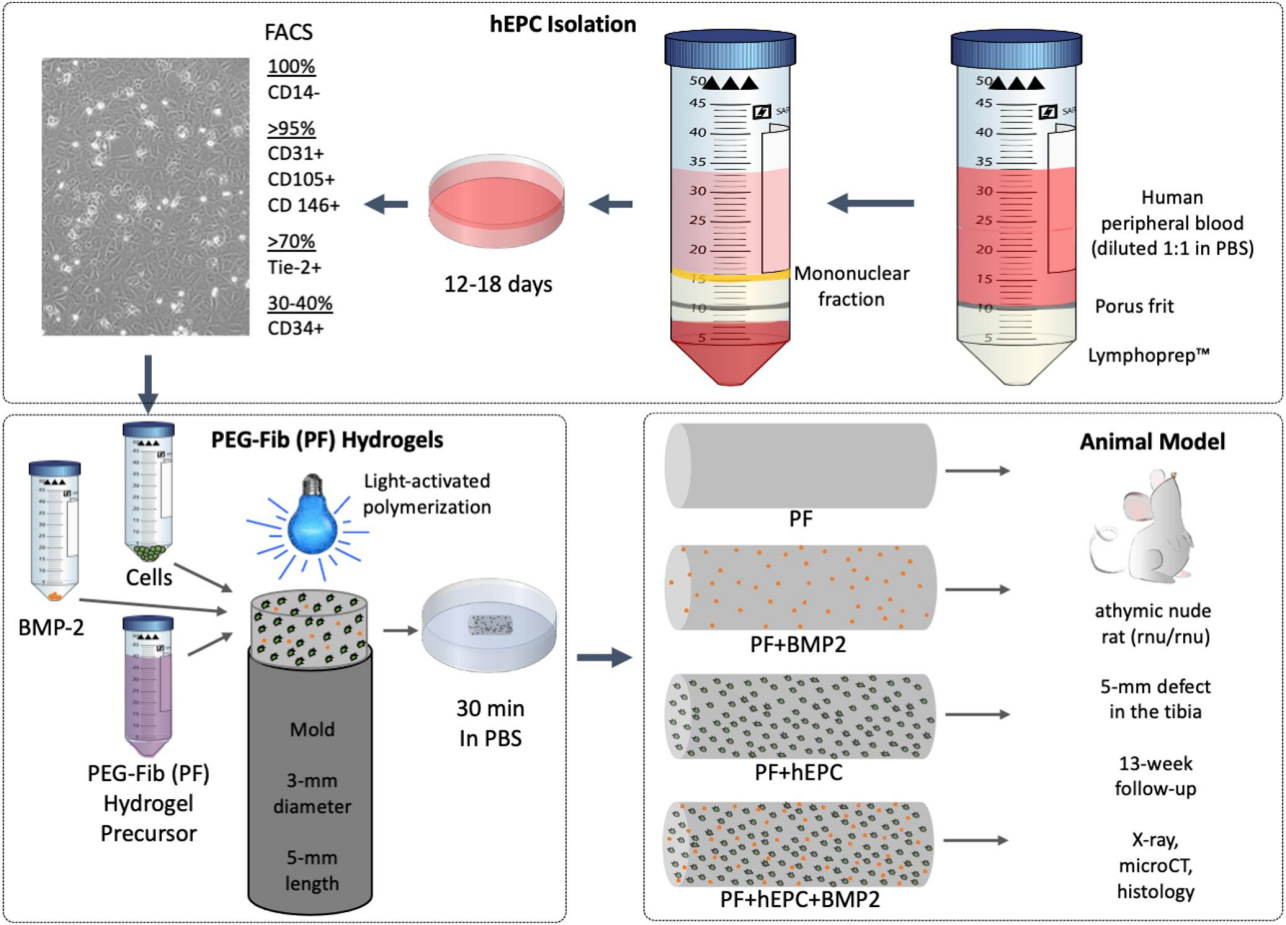
Isolation of Human Endothelial Progenitor Cells (hEPCs), Formulation of Hydrogel Implants, and Animal Study Design. Human peripheral blood samples were processed with a Lymphoprep kit to isolate the mononuclear fraction. The cells were seeded on fibronectin‐coated dishes for up to 18 days. Before their placement in hydrogels, the cells exhibited a cobblestone morphology and were assessed by FACS analysis. Hydrogels were prepared from PEG‐Fib (PF) precursor, with or without hEPCs and/or rhBMP2, placed in a cylindrical mold and exposed to light‐activated polymerization. The hydrogels were pre‐soaked in PBS for 30 min and implanted into the segmental defects in athymic nude mice

Peripheral blood samples were obtained from healthy young volunteers (22–35 years) who signed an informed consent. The individual peripheral blood samples (20–50 ml) were pooled, and hEPCs were isolated as described elsewhere (Rozen et al., 2009; Zigdon‐Giladi et al., 2015). In brief, diluted peripheral blood samples (1:1 with phosphate buffered saline, PBS) were processed using the Lymphoprep™ kit (Axis‐Shield, Oslo, Norway). The mononuclear cell (MNC) fraction was isolated and cultivated on fibronectin‐coated plates (Sigma‐Aldrich, Sleeze, Germany) with EBM‐2 media supplemented with EGM‐2MV SingleQuote (Lonza, Walkersville, MD, USA), containing 20% heat‐inactivated fetal bovine serum, vascular endothelial growth factor (VEGF), fibroblast growth factor‐2, epidermal growth factor, insulin‐like growth factor‐1 and ascorbic acid. Cells were grown at 37°C with humidified 95% air/5% CO_2_. After 4 days, the non‐adherent cells were discarded by gentle washing with PBS, and the cell culture was maintained for up to 2 weeks in complete EGM‐2 medium. The medium was replenished three times per week. Proliferating cells, regarded as EPCs, were observed after 12–18 days (Figure 1) and sub‐cultured by brief trypsinization using 0.5% trypsine/0.2% EDTA (Biological Industries Ltd., Beit Haemek, Israel). These cells were characterized by flow cytometry using antibodies specific for CD105, CD146, CD14, CD34 (mouse anti‐human BD Biosciences, San Jose, CA, USA), Tie‐2 and CD31 (LifeSpan BioSciences, Seattle, WA, USA). Cytometry of the cells was performed by placing them in PBS filled test tubes (5 × 10^5^ cells per tube) and incubating them for 30 min in the antibodies according to the manufacturers' recommendations. After three sequential washings, the cells were re‐suspended in PBS and analyzed using FACScan and CellQuest software (Becton Dickinson & Co., Franklin Lakes, NJ, USA). A Mouse IgG1 FITC isotype (BD Biosciences) was used as a negative control.

### 2.2 Hydrogel preparation

PEGylated denatured fibrinogen (PEG‐Fib, PF) and PEG‐diacrylate (PEG‐DA) were prepared according to published protocols (Appelman et al., 2009). PF hydrogels were prepared from PF with a protein concentration of 8 mg/ml and additional PEG‐DA (3% w/v) as detailed elsewhere (Schmidt et al., 2006). The PF was first mixed with 1.0% (v/v) photoinitiator stock solution made of 10% w/v Irgacure™2959 in 70% ethanol and deionized water. The Irgacure™2959 photoinitiator was generously provided by Ciba Inc. (Basel, Switzerland). The hydrogels were cross‐linked by placing the solution with and without 2.5 × 10^6^ cells/ml hEPCs and/or 7.7 μg/ml rhBMP2 into a mold and exposing it to long‐wave UV light (365 nm, 5 mW/cm^2^) for 5 min at room temperature (RT; Figure 1). The mold had the dimensions of 5 mm length and 3 mm diameter for a total volume of 35 μl. The rhBMP2 was purchased from Medtronic (USA) as part of the INFUSE® kit. The rhBMP2 component of the kit was reconstituted in the provided solution and used immediately or frozen at −80°C for up to 3 months. The frozen rhBMP2 was thawed at room temperature and used immediately to prepare hydrogels.

### 2.3 Segmental tibia defect model

The Technion animal care committee and Institutional Review Board (IRB) reviewed all animal protocols and provided approval in advance for all animal experimentation. Moreover, guidelines set out by the Rappaport Faculty of Medicine of the Technion, Israel Institute of Technology were implemented for all experiments. Male athymic nude rats (Hsd: RH‐FoxN1RNU, Harlan, IN, USA, 13 weeks, 250–300 g) were adapted to cage life for 5 days before surgery. The animals were monitored for weight to ensure stability and proper adaptation. Tap water and regular lab chow were provided ad libitum. Anesthesia was done with Ketamine 10% 90 mg/kg and Xylazine 2% 10 mg/kg IP (Intraperitoneal injection). Pain control was maintained with Buprenorphine 0.1 mg/kg SC (Subcutaneous injection) 10 min before the skin incision and following surgery twice a day for 5 days. The mid‐portion of the right tibia was exposed from the anterior medial side by longitudinal incision (Supplementary Figure S1). An external fixation device consisting of two proximal needles (21GX1 1/2″) and two distal needles (21GX1 1/2″) was placed according to published protocols (Kossover et al., 2020). The needles were fixed using epoxy resin as the external fixations to stabilize the tibia. A 5‐mm gap was made between the proximal and distal needles using a saw, with the ipsilateral fibula left intact. The defects were transplanted with cylindrical‐shaped hydrogels (3‐mm diameter and 5‐mm long) of PF. Four treatment groups were evaluated, including PF hydrogels without cells and rhBMP2 (10 rats); PF hydrogels with 8.75 × 10^4^ hEPCs and without rhBMP2 (5 rats); PF hydrogels without hEPCs and with 0.27 μg rhBMP2 (8 rats); and PF hydrogels with 8.75 × 10^4^ hEPCs and 0.27 μg rhBMP2 (4 rats). The surrounding periosteum was left intact and wrapped around the implants. The underlying fibrous tissue was also wrapped around and sutured with surgical threads to secure the implant in place. The rats were given prophylactic antibiotics Cefazolin 100 mg/kg SC. X‐ray radiographs were taken at 5, 8 and 13 weeks. The rats were allowed relative free ambulation and were sacrificed with CO_2_ at the end of the 13‐week evaluation period.

### 2.4 Digital x‐ray

An Oralix AC Densomat X‐ray machine (Gendex Dental System, Milan, Italy) was used to take radiographs at 5, 8, and 13 weeks after the segmental defect was made. The machine's operating conditions were: 65 kV peak voltage, 7.5 mA anode current, and an exposure time of 0.26 s. Kodak's Dental Imaging Software version 6.5 (Kodak Dental Systems, GA, USA) was used for controlling these parameters.

### 2.5 Micro‐computed tomography (μCT)

Whole rat legs were harvested and scanned using a Skyscan 1076 (Bruker, Belgium) μCT scanner at a resolution of 35 μm, voltage of 100 kV, and current of 100 μA. The defects were then reconstructed, and the extent of bone healing was determined (ConeRecon‐v2.19, Bruker Belgium, Materialize Mimics v14.0, Belgium). Before scanning, 2D images were again collected at 0 and 90° for comparison to the images taken before dissection. In all cases, the samples were loaded centered in the middle of the cylindrical loading tube. A cylindrical region of interest (ROI) measuring 7 mm in length was positioned in the defect for the newly formed bone analysis. The volume of newly formed bone within this ROI was determined using the Mimics software with bone threshold values between 148 and 3056 (arbitrary grayscale unit). The threshold was determined based on visualization of bone morphology, and the same threshold (148‐3056) was rigorously applied to all samples throughout all analyses. Representative 3D‐reconstructions using Mimics software were used to assess the structure and distribution of bone within the defects. For measuring the tissue mineral density (TMD), trabecular number and trabecular thickness, a CTAn (Bruker, Belgium) device was used with a cuboidal ROI 5 mm in length, diameter 6 mm × (4.5–6 mm). An arbitrary threshold of 0–210 was used and the total attenuation coefficient range was set at: 0–255. The TMD was calculated as described elsewhere (Nazarian et al., 2008), calibrated against Bruker Phantom Rods (2 mm diameter). For 0.25 g/cm^3^ CaHA, the attenuation was 0.80013; for 0.75 g/cm^3^ CaHA, the attenuation was 0.59888.

### 2.6 Histology and immunohistochemistry

Rats were euthanized at 13 weeks after the segmental defect was made, and the harvested tissue was fixed in neutral buffered formalin (NBF, 4%). First, the tibia was analyzed by micro‐computed tomography (μCT) and then was sent for histology. Histological preparation involved decalcification in 10% Ethylene Diamine Tetraacetic Acid (EDTA), followed by dehydration in graded ethanols (70%–100%) and finally embedding in paraffin. Serial sections (6‐μm thick) were stained with Hematoxylin & Eosin (H&E; according to manufacturer's protocols; Sigma‐Aldrich) or with immunofluorescence. Briefly, paraffin sections were deparaffinized with xylene, hydrated with a series of alcohol solutions, then boiled for 20 min in 10 mM Citrate Buffer for antigen retrieval. Following permeabilization with 0.5% Triton X‐100, slides were blocked with 5% BSA for 1 h and incubated with Human Lamin A/C Monoclonal Antibody (mab636, Invitrogen. 1:200) overnight in 4°C. Secondary antibody incubation was performed using Goat anti‐Mouse, Alexa Fluor 555 (Invitrogen, Waltham, MA, USA). Finally, slides were stained with DAPI for nuclear detection (Sigma‐Aldrich) and mounted with mounting medium. Stained slides were imaged using a Zeiss LSM 700 confocal microscope. Positive controls included stained samples of human dermal fibroblasts and negative controls included stained samples of rat tissue.

### 2.7 Statistics

Results are reported as mean ± standard deviation (SD). Comparison between two treatments was performed using a Student's *t*‐test. Significant values were set at *p* < 0.05. GraphPad prism analysis software (Version 5.01, GraphPad Software Inc., CA, USA) was used for this analysis.

## 3 RESULTS

### 3.1 Cell isolation and expansion

The hEPCs exhibited a typical cobblestone morphology on the fibronectin‐coated tissue culture dishes and reached confluency within a couple of weeks (Figure 1). FACS analysis revealed that more than 95% of the cells were positive for CD31, CD105 and CD 146. At least 70% of the cells were positive for Tie‐2, and 30%–40% of the cells were positive for CD34. All cells were negative for CD14 (a haematopoietic cell marker), ruling out their monocytic origin and supporting their angioblastic origin.

### 3.2 In vivo evaluation

Each animal was examined immediately after the procedure and before each X‐ray imaging time‐point as well as at the time of sacrifice. The post‐operative response was uneventful, as all animals survived the procedure and the duration of the experimental follow‐up. The animals did not show signs of infection, nor were there any incidents of chronic inflammation in the operated animals (Supplement Figure S2). Similarly, the weight of each animal was stable during the 13‐week follow‐up, indicating that there were no adverse events related to the physical health of the animals.

### 3.3 New bone formation

**FIGURE 2:**
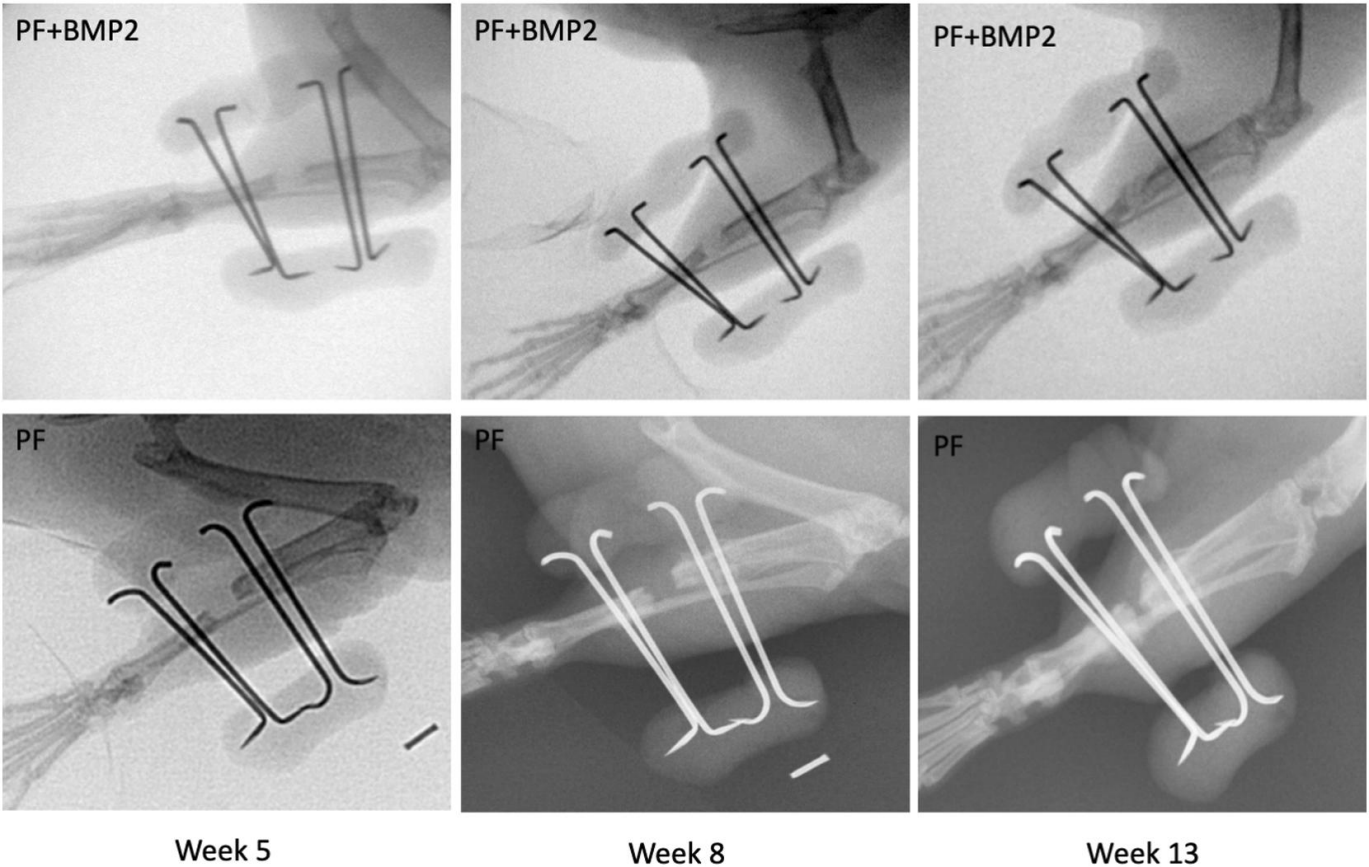
Radiographs showing new bone formation in treated rats after 5, 8 and 13 weeks. New bone bridging the entire defect was evident after 13 weeks when the formation of a periosteal callus was apparent by week 5 (e.g., PF + BMP2 treated animal). In the absence of a periosteal callus, the extent of osteoneogenesis was limited to the partially regenerated bone in the gap (e.g., PF treated animal)

Radiography was used to identify evidence of de novo bone formation in the segmental defects of the tibiae during the experimental follow‐up period. The observations from week five identified osteogenesis in the formation of a periosteal callus in some treated animals, namely those implanted with the PF + BMP2 + hEPC and PF + BMP2 hydrogels (Figure 2). Conversely, there were treated rats that lacked any sign of radiographically detectable bone in the defects after 5 weeks, most notably those implanted with PF and PF + hEPC hydrogels. When a periosteal callus was present, the osteogenesis continued over time, becoming more apparent by week 8 and culminating in a total boney bridge of the defect by week 13. In the absence of a callus formation, the segmental defect remained unabridged and nonunion was evident by week 13. Representative radiographs of rats implanted with either PF or PF + BMP2 are shown in Figure 2 to demonstrate the follow‐up evaluation of nonunion or complete boney bridging of the defects, respectively.

**FIGURE 3:**
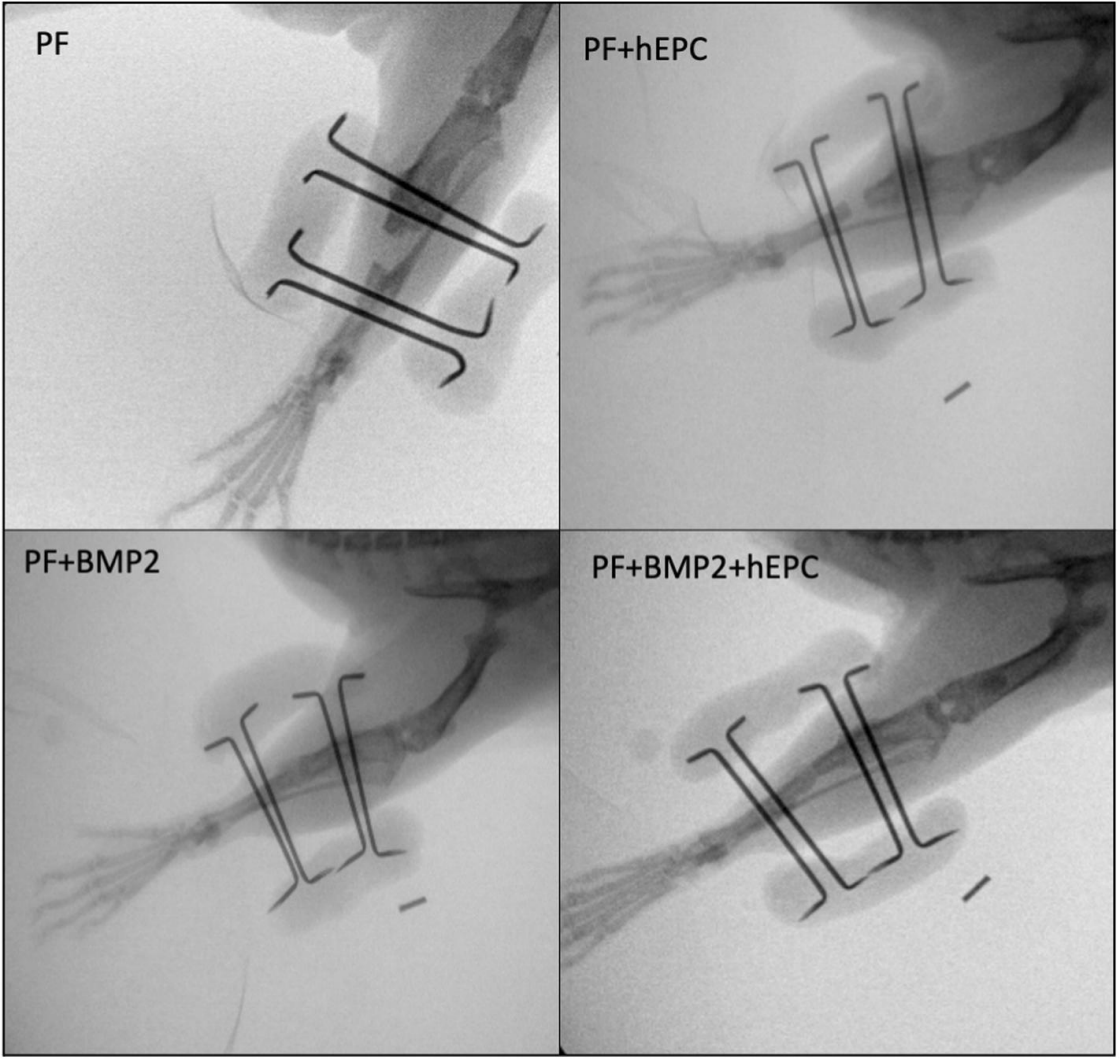
Representative radiographs for each of the different treatments in the study after 13 weeks. Total bridging of the defect was typically observed in the PF + BMP2, and PF + BMP2 + hEPC treated rats. Partially regenerated bone was usually observed in PF, and PF + hEPC treated rats. An osteogenesis response near the fixation pins of the external fixators was evident in some of the treated rats, irrespective of their treatment

Several patterns were evident when comparing radiographically detectable bone in the defects of the different treatments at week 13. For example, PF and PF + hEPC treated defects rarely exhibited a full boney bridging of the defects, whereas nearly all the BMP2‐treated defects (with and without hEPCs) were fully bridged with new bone by 13 weeks (2 of 12 treated defects were not fully bridged). Representative radiographical evidence of this pattern is presented in Figure 3. The results indicated that the BMP2‐treated defects exhibited the most extensive and widespread osteoneogenesis in and nearby the defect site. In contrast, non‐BMP2 treated defects exhibited little osteogenesis, and much of the new bone formation at 13 weeks was only near the defect margins. Some of the non‐BMP2 treated defects showed small radiographically opaque regions within the defect margins. This indicated pockets of new bone formation without a boney bridging of the defect. There was also a strong osteogenesis response to the fixation pins of the external fixators, which was evident radiographically in some of the animals, irrespective of their hydrogel treatment.

### 3.4 μCT analysis

**FIGURE 4:**
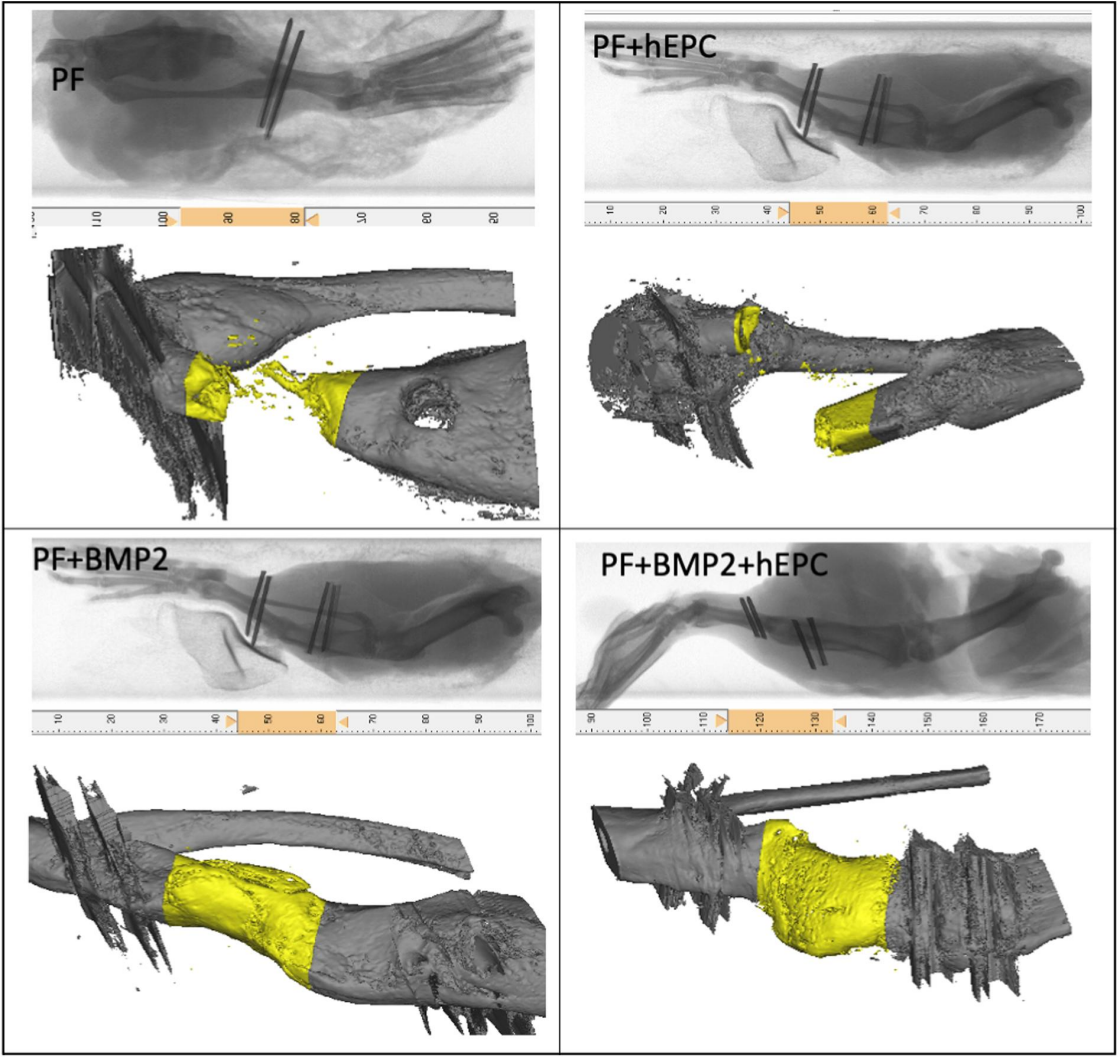
Representative μCT images for each of the different treatments in the study after 13 weeks. The respective X‐ray of the rat hind‐limbs (shown) was used to ensure proper orientation of the bone during the imaging procedure after the removal of the external fixation device. Total bridging of the defect margins (highlighted in yellow) was typically observed in the PF + BMP2 and PF + BMP2 + hEPC treated rats, whereas partially regenerated bone was usually observed in PF and PF + hEPC treated rats

**FIGURE 5:**
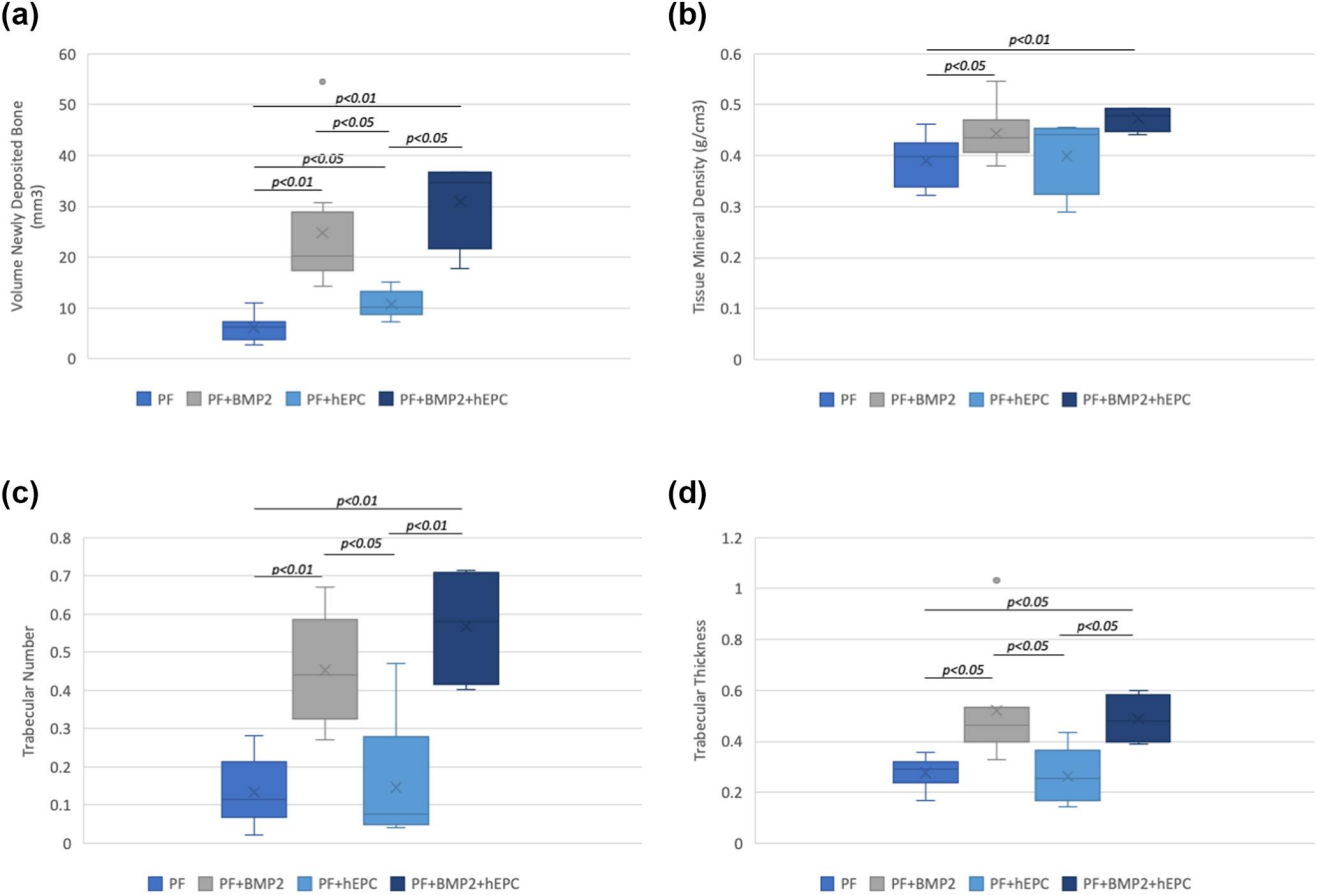
Graphical representations of quantitative μCT analysis data of the four independent treatments in the study design. (a) The volume of newly deposited bone within the defect margins was calculated based on the μCT raw data. (b) Tissue Mineral Density (TMD) characterizes the bone formation in terms of the mineralization within the defect margin (calibrated against Bruker 2 mm diameter phantom rods). (c) Trabecular number is a measure of the average number of trabecule per unit length (mm^−1^). (d) Trabecular thickness is the mean thickness of trabecule (mm), assessed using direct 3D methods. Significant differences as determined using student's *t*‐test are indicated directly on the graphs by presenting the *p*‐values (*p* < 0.01 and *p* < 0.05). Differences that were not statistically significant (*p* > 0.05) are not indicated on the graphs

μCT analysis was performed on the harvested defects to quantify the quality of new bone formed in each treatment group (new bone volume, tissue mineral density, trabecular number and trabecular thickness) as per established guidelines (Nazarian et al., 2008). The external fixators were removed by cutting off the epoxy from the metallic pins in the tibiae just before μCT analysis. X‐ray images of the legs without the epoxy fixators were obtained prior to μCT imaging, in order to arrange the anatomical orientation of the defect in the μCT chamber. The μCT data confirmed the radiographical observations that the PF + BMP2 and PF + BMP2 + hEPC treated defects exhibited the highest volumes of bone tissue compared to the other treatments (Figures 4 and 5). Quantitative analysis of the reconstructed images of the μCT scans revealed that the addition of rhBMP2 results in a statistically significant increase in new bone volume in the defect margins (*p* < 0.05). Specifically, there was a 4‐fold increase in new bone volume in the PF + BMP2 treated defects compared to the PF treated defects (Student's *t*‐test, *n* ≥ 8, *p* < 0.05). Similarly, there was nearly a 3‐fold increase in the new bone volume of the PF + BMP2 + hEPC treated defects compared to the PF‐hEPC treated defects (Student's *t‐*test, *n* ≥ 4, *p* < 0.01; Figure 5). The quantitative μCT data also indicated a near 2‐fold increase in new bone volume due to the presence of hEPCs in the non‐BMP2 hydrogel defects (Student's *t‐*test, *n* ≥ 5, *p* < 0.05). However, this increase in new bone volume due to the presence of hEPCs was less pronounced in the BMP2‐containing hydrogel treatments (Student's *t‐*test, *n* ≥ 4, *p* > 0.05). Similar trends were observed with the other measured parameters of the bone in the defect margins, including tissue mineral density (Figure 5b), trabecular number (Figure 5c) and trabecular thickness (Figure 5d). Table 1 summarizes the mean values of each parameter, including the standard deviation of the mean.

**TABLE 1:** μCT data analysis results showing the mean and standard deviation from the mean

Treatment	Volume of new bone (mm^3^)	Tissue mineral density (g/cm^3^)	Trabecular number (mm^−1^)	Trabecular thickness (mm)
PF	6.1 ± 2.4	0.39 ± 0.05	0.13 ± 0.09	0.28 ± 0.06
PF + BMP2	24.8 ± 13	0.44 ± 0.05	0.45 ± 0.15	0.52 ± 0.22
PF + hEPC	10.8 ± 2.8	0.40 ± 0.07	0.15 ± 0.18	0.26 ± 0.11
PF + BMP2 + hEPC	31.0 ± 8.9	0.47 ± 0.02	0.57 ± 0.16	0.49 ± 0.10

### 3.5 Histological evaluation

**FIGURE 6:**
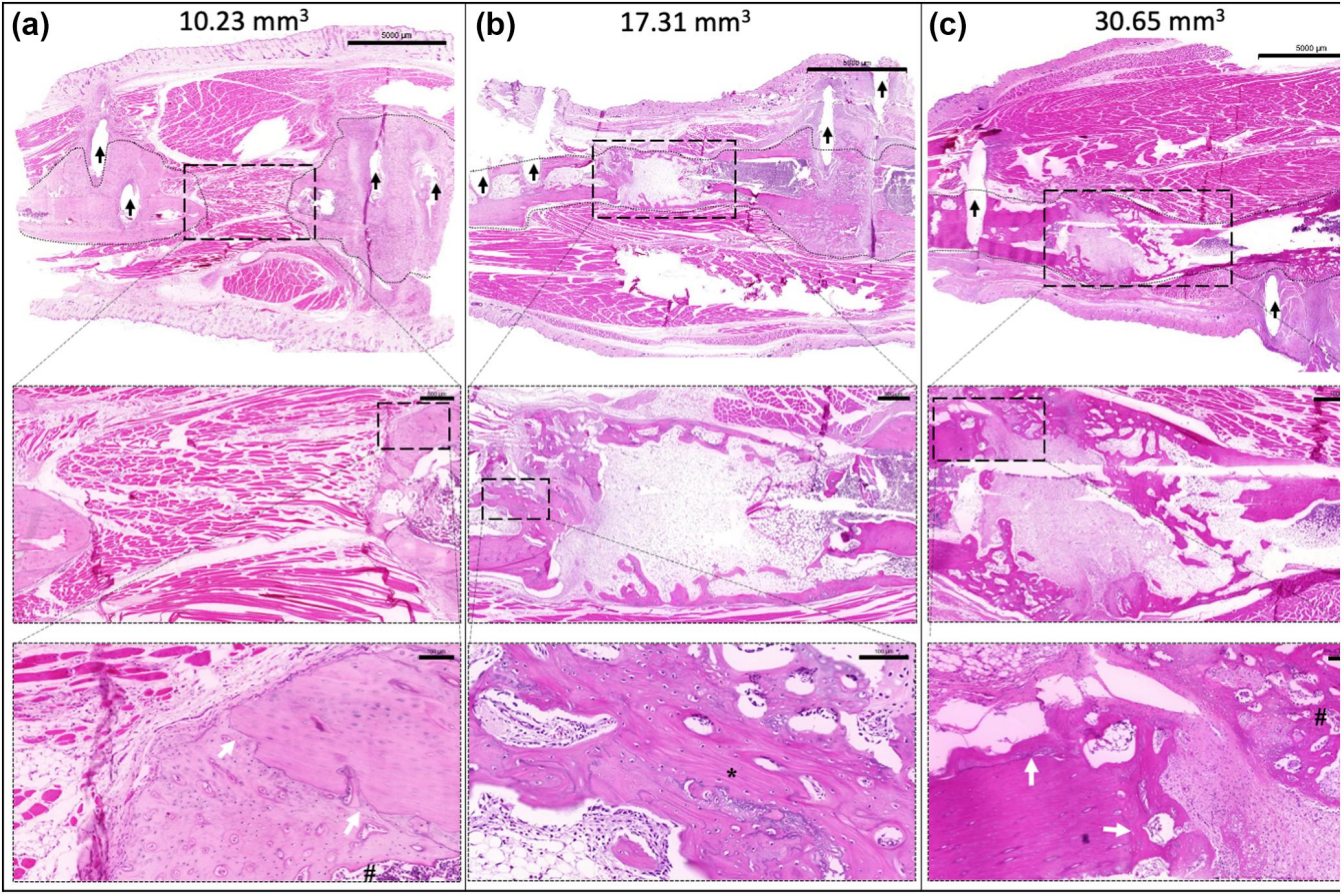
Representative histological analysis for the different treatments in the study after 13 weeks (H&E staining). The longitudinal sections of the hydrogel‐treated tibial defects show partial to complete bone regeneration in the defect site. The location of the defect is indicated by the large rectangular box and the bone tissue is highlighted by a dashed line. The external fixation pin locations are indicated by the black arrows. The extent of osteogenesis varied from non‐union with partial regeneration in the PF‐treated animals (a), to full bridging of the defect in the BMP2‐treated animals (b, c). Higher magnification regions of interest show evidence of osteogenesis within these images, including lamellar‐fibered compact bone (indicated by *) and a Haversian system (indicated by #). The interphase between the pre‐existing cortical bone and the new bone formation is indicated by the white arrows. The bone volume for each specimen is provided at the top of the image for reference. High resolution versions of these images can be found in the supplementary data. Top row scale bars equal 5000 μm for (a–c); Middle row scale bars equal 500 μm for (a) and (b), and 1000 μm for (c) Bottom row scale bars equal 100 μm for (a) and (b), and 200 μm for (c)

Histological analysis following μCT confirmed the observed trends in the bone repair results. Complete boney bridging of the defect was observed in the PF + BMP2, and PF + BMP2 + hEPC treated rats, with osteons and a lamellar fibered organization. Newly formed bone was observed from end‐to‐end of the osteotomies. This bone mainly consisted of birefringent, lamellar‐fibered, compact type bone. There was also a Haversian system with a moderate number of vessels within. Nonunion was primarily observed in the PF, and PF + hEPC treated animals (Figure 6), with osteoneogenesis evident at the endosteal and subperiosteal aspects in some of these treated animals. Endochondral caps in these animals were also occasionally seen, with characteristic hypertrophic chondrocytes present. Consequently, in the nonunion defects, cartilaginous islands with endochondral ossifications were observed. In all defects, the PF implants were almost entirely resorbed, and any new bone was seen in the implant locale. The presence of implanted hEPCs, as evaluated by immunofluorescence staining for human Lamin A/C, was not evident in the defect after 13 weeks based on the lack of positive‐stained cells in any of the hEPC‐treated samples (data not shown).

## 4 DISCUSSION

The routine use of autologous bone grafting to repair long bone defects is hampered by donor site morbidity and limited availability. Alternatives to autologous bone grafting include allografts and off‐the‐shelf bone fillers such as β‐tricalcium phosphate (βTCP). Despite some success for these products, they are often met with inadequate efficacy and other limitations, including concerns of disease transmission in the case of implants made from allogeneic tissues. For these reasons, clinical treatment of long‐bone critical‐sized defects has turned to the use of exogenous biomaterials, autologous cells, genes, recombinant growth factors and their combinations. For example, autologous cell‐based strategies facilitate more rapid osseous repair as compared to acellular approaches (Lim et al., 2019). Bone morphogenic proteins such as rhBMP2 can accelerate osteogenesis (Murali et al., 2013) without autologous cells (Gruber et al., 2014; Jung et al., 2008; Smith et al., 2014; Wen et al., 2016). Gene delivery can also be used to increase the local concentration of osteogenic factors in the bone defects (Manaka et al., 2011), including with the use of genes encoding BMP2 (Hsiao et al., 2016). The combined use of osteogenic factors and certain cell therapies appears to act synergistically on the bone repair process (Jo et al., 2013; Kim et al., 2007). There are still issues that complicate using such advanced combination approaches for long‐bone repair, most notably safety concerns. Nevertheless, given the limited efficacy of alternative therapies for treating segmental bone defects, the use of cells, growth factors, or gene therapy are necessary in this clinical indication.

Biomaterials‐based strategies for bone repair have recently been applied to address some of the concerns in long‐bone regeneration using combination devices (Jung et al., 2009; Wen et al., 2011). Although these materials have demonstrated some clinical efficacy as stand‐alone implants (Wang et al., 2005) or as implants with pre‐defined bioactivity (Peled et al., 2007; Thoma et al., 2014), the vast majority of biomaterial‐based approaches take advantage of osteogenic factors and/or cells to accelerate the bone regeneration (Le et al., 2019). For example, hydrogel biomaterials made from polymers such as PEG can potentially reduce the safety concerns of supraphysiological rhBMP2 doses by providing a controlled release depot for the factor, thereby reducing the overall concentration in the implant device (Thoma, Jung, et al., 2017; Thoma, Weber, et al., 2017). PEG‐based hydrogel biomaterials have also been used as a delivery vehicle to localize osteogenic cells at the implant site and enhance the osteogenic potential of these cells for prolonged durations (Aziz & Bryant, 2019). In the context of a segmental bone injury, we and others have shown that such hydrogels can improve the complete bridging of the rat tibia defect model when combined with exogenous growth factors (Ben‐David et al., 2013; Kossover et al., 2020). Wojtowicz et al. used polycaprolactone (PCL) coated with a synthetic osteogenic collagen‐mimetic GFOGER triple‐helical peptide to enhance bone formation in a segmental defect in the rat femur (Wojtowicz et al., 2010) based on the peptide's ability to bind to the alpha2 beta1 integrin receptor. The peptide was also used with <50 μg/ml of rhBMP2 (Shekaran et al., 2014), demonstrating better outcomes when compared to the typical collagen sponge scaffolds. This synthetic GFOGER peptide was also introduced into a biodegradable PEG‐based hydrogel, improving the efficacy of the implant in a radial defect model. Another biomaterials‐based approach was applied with synthetic peptide amphiphile nanofibers to stimulate the signaling cascade of BMP2. This peptide, which binds heparin, was immobilized on a collagen sponge scaffold together with low concentrations of BMP2. The immobilization resulted in improved osteogenesis in a rat critical‐size femoral defect (Lee et al., 2013). Beyond long‐bone repair, these biomaterials have been used in maxillofacial osteogenesis applications. For example, a biodegradable triblock PEG‐PCL‐PEG copolymer (PECE), collagen and nano‐hydroxyapatite (n‐HA) was successfully applied to stimulate bone formation in vivo using a cranial defect model in the rabbit (Fu et al., 2012).

In this study, we use a PF biomaterial to deliver a combination of hEPCs and rhBMP2 to a segmental bone defect in the rat tibia. This material has a proven track record in terms of being non‐toxic and biocompatible with multiple cell types (Fuoco et al., 2014, 2015; Plotkin et al., 2014; Rufaihah et al., 2013, 2017). The PF is made of components considered safe by the FDA for other clinical indications (Almany & Seliktar, 2005; Dikovsky et al., 2006; Thompson, 2009; Torchiana, 2003). It has been used extensively in biomedical research and development, most notably as a tissue repair device called GelrinC used to treat focal cartilage injuries in human patients (Trattnig et al., 2015). In terms of biodegradation, the PF can be proteolytically degraded in vivo by cellular proteases and hydrolysis. Control over biodegradation rate is afforded by the additional crosslinking of the hydrogel using PEG‐DA in the implant formulation (Dikovsky et al., 2008). Accordingly, the PF composition in this study was based on our previous studies showing that the implant was completely removed from the defect site by cell‐mediated proteolysis within the timeframe of the 13‐week follow‐up (Peled et al., 2007). Using this cross‐linking density also ensured that the hydrogel entraps the growth factor (Frisman, Orbach et al., 2010; Frisman, Seliktar et al., 2010, 2011, 2012) because of the small mesh size (Bearzi et al., 2014; Berdichevski et al., 2015; Mironi‐Harpaz et al., 2014; Sarig‐Nadir & Seliktar, 2008; Terraciano et al., 2007; Testa et al., 2017; Weitzenfeld et al., 2016; Xu et al., 2018; Xu et al., 2015). Previous studies have shown that the molecular structure of the PF scaffold is amorphous and likely to entrap rhBMP2 within the network (Schnabel‐Lubovsky et al., 2019). The factor can then be released for several weeks (Dror Ben‐David et al., 2013), synchronized with the in situ degradation of the material (Peled et al., 2007). Our hypothesis stipulates that the cell‐laden PF implant will expedite bone repair in a segmental critical size defect compared to acellular PF implants. We also examine the combination of cell therapy and sustained release of a relatively low concentration rhBMP2 (7.7 μg/ml); its release being mediated by the material degradation (Metzger et al., 2016). The low concentration of rhBMP2 used in this study was chosen based on our previous experiments showing the benefits of controlled release in reducing the need for supraphysiological levels of growth factor in bone repair indications (Ben‐David et al., 2013). We compared four treatments, including with and without hEPCs and with or without rhBMP2. All materials that were used were made with the same composition of PEG and fibrinogen. Consequently, the PEG is considered inert and cannot stimulate bone repair (Bikram et al., 2007). We have previously shown that the fibrinogen in the material could improve bone repair, particularly as related to its role in the natural tissue repair process (Thompson, 2009).

The use of hEPCs was motived by previous studies showing increased vasculogenesis associated with the cellular implants can enhance bone regeneration in a rat calvarium model (Zigdon‐Giladi et al., 2013, 2015). Both human and rat EPCs, when mixed with βTCP, resulted in a significant increase in vasculogenesis and bone volume compared to βTCP controls. In the present study, we used athymic nude rats as the animal model to avoid immunogenic rejection of transplanted human cells. The hEPCs exhibited the typical cobblestone morphology and specific surface antigens that characterize EPC (Hirschi et al., 2008; Hur et al., 2004; Yoder et al., 2007). EPCs originate in the bone marrow; however, these cells are isolated from the peripheral blood because they are recruited by a gradient of vasculogenic/angiogenic molecules (e.g., VEGF, erythropoietin; Ferguson et al., 1998). Asahara et al. were the first to describe their isolation from peripheral blood (Asahara et al., 1997). Consequently, hEPC has been used to treat ischemic tissue after acute myocardial infarction (Isner & Losordo, 1999; Kalka et al., 2000) and have been explored for treating unstable angina, stroke, diabetic microvasculopathies, pulmonary arterial hypertension, atherosclerosis, and ischemic retinopathies (Jung & Roh, 2008; Rafii & Lyden, 2003; Sekiguchi et al., 2009; Tateishi‐Yuyama et al., 2002; Ward et al., 2007).

The study results demonstrate that hEPCs improve overall skeletal regeneration in the segmental bone defect in nude rats. The μCT data shows a near two‐fold increase in new bone volume in the PF + hEPC group compared to the PF control group. The use of rhBMP2 in the PF was much more beneficial to bone regeneration, with a four‐fold increase in new bone volume evident in the PF‐rhBMP2 group, compared to the control PF group. These results agree with our previous studies using PF and similar concentrations of rhBMP2 in a wild‐type rat segmental defect model (Kossover et al., 2020). Combining rhBMP2 and hEPCs did not result in higher levels of new bone formation when compared to PF + BMP2 treatment. Taken together, it appears that any benefit of the hEPCs in this study is overshadowed by the very high efficacy of the rhBMP2 treatment. This may be explained by the lack of hEPCs present in the defect locale at 13 weeks, which suggests that the cells do not persist in the implant to contribute sufficiently to the new bone formation beyond what is already being stimulated by the controlled release of the rhBMP2.

Other studies have shown benefits to using hEPCs in different bone regeneration models, most notably in a segmental defect in sheep tibia (Rozen et al., 2009). They filled a 3.2 cm defect in the sheep tibia with autologous sheep EPCs 2 weeks after the initial injury. They showed complete bridging of the gap in nearly all defects treated with the EPCs after 12 weeks post‐transplantation. Consequently, non‐union was observed in the non‐treated animals. Another study in the rat femur used rat EPCs in a collagen scaffold and implanted this into a 5 mm segmental defect (Atesok et al., 2010). They showed all EPC‐treated animals with complete bridging of the defect, whereas control‐treated animals resulted in non‐union. Seebach et al. used hEPCs on βTCP scaffolds to treat segmental bone defects in rat femur (Seebach et al., 2010). They documented an increased vascularization of the graft 1 week following EPC transplantation and improved osteogenesis after 12 weeks compared to βTCP and MSC controls. With the apparent benefit to bone regeneration indicated in these studies, the exact role of EPCs must be further clarified. In terms of the scaffold contributing to the observed bone regeneration in this study, the PEG is not likely to play a critical role in osteogenesis (Humber et al., 2010). Others have studied PEG hydrogels in bone repair models and showed that the PEG‐only hydrogels cannot regenerate bone in the defects (Brockmeyer et al., 2015). PEG hydrogels have been shown to be a good material for the entrapment and sustained delivery of osteoinductive factors (Bikram et al., 2007; Burdick et al., 2002; Catros et al., 2015; Ma et al., 2016). Nevertheless, the dense PEG hydrogel, which degrades rather slowly by hydrolysis, can remain intact and thereby impede the regeneration process. The biodegradation pattern of the PF hydrogel, on the other hand, has been studied previously in a segmental defect model and shown to coincide well with the time‐frame of the bone regeneration process. The PF hydrogels were also evaluated for histocompatibility within the site of the tibial defect, where they were found to be partially to fully degraded after 13 weeks. When they remained in the defect, they were always surrounded by a foreign body granulation tissue and appeared completely acellular. This granulation tissue contained fat cells with a few inflammatory cells. Macrophages and lymphocytes were also found near the hydrogel remnants. We speculate that these PF hydrogels were undergoing phagocytic biodegradation by granulation tissue, consistent with our previous studies (Dror Ben‐David et al., 2013; Kossover et al., 2020; Peled et al., 2007).

One of the limitations of the current study is that we did not evaluate the exact mechanism of action associated with the enhanced osteogenesis of the combination hEPC and rhBMP2 treatment. Others have documented the role of several growth factors in mediating cellular crosstalk between endothelial cells and osteoprogenitors in bone repair, including VEGF, BMP2 and transforming growth factor‐beta (TGFβ), as well as direct cell‐cell interactions (Fuchs et al., 2009). It is possible that the rhBMP2 facilitated the enhanced EPC‐mediated osteogenesis through a similar crosstalk mechanism. This synergy with the hEPCs and rhBMP2 in bone regeneration must be further explored. For example, the hEPC response may be enhanced using more than a single growth factor in the scaffold. Some recent studies have investigated enhanced bone repair using a combination of two or more growth factors that are released from hydrogel scaffolds (Bachl et al., 2009). The delivery of both TGFβ3 and BMP2 enhanced bone repair in a chick femur model (Smith et al., 2014). This study used an organotypic culture system to demonstrate that TGFβ3/BMP2 was most potent for bone repair. Scaffolds containing BMP2 and fibroblast growth factor‐2 (FGF‐2) were used to underscore the indispensability of BMP2 in the process of bone regeneration (Charles et al., 2015). There are also studies that highlight the important role of vascularization in osteogenesis, with or without angiogenic cell therapy. For example, the use of pro‐angiogenic growth factors delivered by hydrogel scaffolds can facilitate much better vascular network formation and thus contribute to the bone repair process (Blache et al., 2016; Garcia et al., 2016).

Another limitation of the study is the lack of an empty control group. We cannot preclude that some de novo bone formation observed in the defects may be attributed to spontaneous healing. However, spontaneous bone formation in this type of critical‐sized bone defect and animal model is not expected to occur (Brockmeyer et al., 2015; Peled et al., 2007). To fully substantiate the results, further studies that include an empty control group should be performed. The relatively few animals also limited the study in the cellular treatment groups. This limitation should be addressed in future experiments with larger groups for the PF + hEPC and PF + BMP2 + hEPC treatments. If the hydrogels used in this study take longer than 13 weeks to be fully resorbed in the defect site, future studies should also evaluate a follow‐up duration that is consistent with the complete resorption of the implant material. This is particularly critical if the un‐resorbed hydrogel obstructs the repair tissue formation in the defect margins. Nevertheless, the amount of bone formation observed in the PF + BMP2 + hEPC treated animals was far more extensive, thereby corroborating the role of the relatively small amount of factor and the hEPCs in the observed repair process. Finally, the segmentation of the bone defect region is another limitation of the current study that may have contributed to minor errors in the quantitative μCT bone metrics. Specifically, given the proximity of the defect to the tibiofibular junction, the bone tissue in the fibula had to be manually excluded in some cases from the region analyzed by μCT. However, these adjustments were minor and were guided by visual identification of a radiolucent gap between the newly formed bone tissue and the periosteal surface of the fibula.

## 5 CONCLUSIONS

Different hydrogel formulations were tested to assess long‐bone repair using delivery of 7.7 μg/ml rhBMP2 and/or hEPCs. Hydrogels containing the rhBMP2 protein, with or without hEPCs, could bridge the 5‐mm gap in the tibia with new bone formation, concurrently to their resorption from the defect locale. Hydrogels containing rhBMP2 and hEPCs performed the best in terms of new bone volume in the defect site. Hydrogels without hEPCs or rhBMP2 were significantly inferior in their capacity for osseous regeneration as measured by bone volume in the defect margins. We, therefore, conclude that the presence of hEPCs and 7.7 μg/ml rhBMP2 can accelerate the repair process after 13 weeks when delivered from a biocompatible and biodegradable matrix.

## CONFLICT OF INTEREST

The authors have declared that there is no conflict of interest.

## AUTHOR CONTRIBUTION STATEMENT

Talia Cohen planned and performed the rat tibia implantation experiments and x‐ray assessments. Olga Kossover performed the histological analysis and prepared samples for microCT. Eli Peled designed the rat model surgical procedure and planned tibia implantation experiments. Tova Bick performed the human cell isolation procedure. Lena Hasanov prepared the hydrogels for implantation. Tan Tuan Chun performed the microCT analysis. Simon Cool designed the microCT procedure and reviewed the manuscript. Dina Lewinson designed the cell isolation protocol, planned the implantation experiments and reviewed the manuscript. Dror Seliktar designed the experimental protocols, planned the experiments and wrote the manuscript.

## Supporting information

Supporting Information S1

## Data Availability

The data that support the findings of this study are available from the corresponding author upon reasonable request.
