# Supplementary material for: A combined cell and growth factor delivery for the repair of a critical size tibia defect using biodegradable hydrogel implants
Source: J Tissue Eng Regen Med. 2022 Feb 4;16(4):380–95. doi: 10.1002/term.3285 (PMC9303443; doi:10.1002/term.3285)
Supplement: Supplementary file 1 — Supporting Information S1 [file TERM-16-380-s001.docx]

Supplementary Data for Cohen Et al. 2021

**Supplementary Figure 1:** Animal care and surgical procedure

**Supplementary Figure 2:** High resolution images of histological analysis for PF-treated specimen with a bone density of 10.23 mm^3^.

**Supplementary Figure 3:** High resolution images of histological analysis for BMP2-treated specimen having a bone density of 17.31 mm^3^.

**Supplementary Figure 4:** High resolution images of histological analysis for BMP2-treated specimen having a bone density of 30.65 mm^3^.
